# The future of health care in Croatia

**DOI:** 10.3325/cmj.2011.52.433

**Published:** 2011-06

**Authors:** Nataša Škaričić

Most of the time, I criticize the moves of the health administration or write about the scandals in the field, but rarely I discuss possible solutions for the catastrophic situation in the health care system. If at a conference or in a TV program I am asked about it, to save the time I stick to the political reality and claim that I see no solutions since, without nitpicking, I cannot name a single one among the Croatian politicians who could make some serious changes in the system. All our politicians dealing with health care have either already had their turn in the governing position in health care, have not shown enough competence or interest in the health care issues, or are themselves part of the dysfunctional health care processes (mostly all of these possibilities). Since the system’s destiny depends solely on them because the profession and ethics already atrophied, chances for any serious change are slim.

Is this too rough an estimate?

Unfortunately no, since only a succession of completely incompetent governing officials and a lack of all critical reasoning have brought us into this situation.

Let me tell you an anecdote that says a lot. A few months ago, I was invited by a small political party, whose members I respect a great deal so I would not like to name anyone, to join their health committee. I said that I could only serve the function of a professional consultant since I do not see myself as a member of any party. This proposal was immediately rejected as they generally do not want a professional consultant. Then I, out of curiosity, asked what their program was and got a reply that they did not have any program yet but that they presumed that we were on the same side since I had written a lot against Andrija Hebrang, a politician from the longest ruling, right wing political party, Croatian Democratic Union (HDZ).

This is a paradigmatic example since it says a lot how health care issues are perceived in Croatia. First, I have never written ‘against Andrija Hebrang’, but just criticized his moves in the health care system and wrote about some corruption scandals he was involved in. This has nothing to do with health care reforms, only that some policies that were introduced by Hebrang as the Minister of Health should have been changed. Since then, many processes took place that have nothing to do with Hebrang and would have been impossible without a whole succession of incapable individuals. Besides that, how can anyone create a serious health care reform solely on the negation of one person’s work? What does my reporting on Hebrang’s work have to do with any party’s health care program? If Hebrang’s name is a synonym for health care centers’ devastation or confusion that was created about physician’s dual practice, is it then enough to be ‘against Hebrang’ or do you have to have a sound system analysis, package of measures, and forecasts of results to be able to discuss what should be done next.

I am afraid that the Croatian most powerful opposition party, Social Democratic Party (SDP), has a similar approach. Since the left-wing opposition will most probably win the next elections, now it is time to start discussing their program. They do not have much to say about health, same as the party that invited me to become their member: they have a short history (one mandate since 2000), a few sentences form the party’s economy program, power-point presentation on the situation in health care (1), and a ministerial candidate, former assistant minister, Dr Rajko Ostojić.

SDP and its coalition partners in 2000 began making changes in the health care system also on the wings of ‘antihebrangism’, with the minister Ana Stavljenić Rukavina. Their most important idea was to regulate, even ban, physicians’ dual practice in private and public sector allowed by Hebrang, since this kind of work without precise regulations could lead to a grave conflict of interest. To say it simply: if you allow public physicians to work in their private practices in the afternoon (in 1994 this was allowed only to university teachers of medicine), and do not control the contract details, their earnings, and work-norm at their workplace, you get the effect of ‘patients relocation’ to the private sector, which is more profitable for the physician. This regulation has done a lot of damage to the Croatian public health sector (2).

The wish of SDP and its coalition to solve this problem is praiseworthy, the more so because in that moment it was a truly revolutionary move, but they neither made any serious analysis of this practice nor eventually changed the law – for a while, physicians were allowed to work in the afternoon only in public hospitals, which made them go to their first massive strike, so everything was back at the beginning. To be more precise – everyone was allowed to have dual practice, not only university teachers of medicine.

SDP government did make the financial management of the system more transparent, brought out some important issues such as physicians’ relationship with the pharmaceutical industry, presented the first relevant results of the hospital management analysis, organized the first work inspections, and even pressed charges against some physicians, but that was all.

In their current programs, health is mentioned only in the scope of economy projects – economy experts proposed that to increase the competitiveness and flexibility of the work market, health care contributions should be reduced (3), although they did not have any forecasts on how this would affect the system and if it was feasible at all since we have the least transparent health costs. Since the Croatian Institute for Healthcare Insurance in years has not issued an annual financial report on health institutions, we are left to see how SDP experts will reduce health care contributions when they see the real financial situation in the sector.

Finally, when Dr Rajko Ostojić becomes a health minister he will have substantial difficulties with the media. He is the former assistant to the minister Stavljenić Rukavina, who resigned because of the Baxter scandal (3), when dozens of dialysis patients died in Croatia. Ostojić was her right hand and as soon as the coalition appointed her successor, Andro Vlahušić from HNS, Ostojić also resigned (4), which at the time was considered the right thing to do. How will the media react when someone who already left a high position in the Ministry of Health assumes an ever higher position? Also, Ostojić was mentioned in the context of two unpleasant corruption scandals, the scandal around the acquisition of CT equipment for the University Hospital Center Zagreb (5), and the Pegassys scandal (6), in which his potential conflict of interest in placing a hepatitis medicine on the subsidized medicines list was questioned.

None of this has been cleared out yet and it has not been proven that Ostojić has ever done anything wrong, however all that is remembered about persons involved in all Croatian scandals is that there was something murky about them. If SDP’s image is based on being an antipode to HDZ, choosing this person to be the Minister of Health is not the wisest move. Besides that, Dr Ostojić does not have a health care program, which is the biggest problem.

This is why I will go back to question from the beginning of this text: what exactly has to be done for the Croatian health? Not taking into consideration all the data that are currently not accessible, I find the following measures the most important.

First, it is necessary to start discussing the necessity of sustainability of the public health system, regardless of the extra sources of funding. This discussion should be separated from the discussion on other issues related to the social contract change in the transition from socialism to the so-called liberal capitalism, since observing health in the context of market economy is a utter nonsense – the state is obligated to provide health care services for all its citizens, and guarantee maximum freedom on the private health market, and that is all. These two concepts are not mutually exclusive.

If we agree that public health in every civilized country is based on the principles of solidarity and that the right to health care is one of the basic human rights, we will start with defining the rules for the budget redistribution rather than question the sustainability of the public health sector. This means that the state should know what exactly it pays for and make transparent decisions on what has to be publicly funded and what has to be left to the private investment.

Second, it is necessary to destroy the myth of irrational health care spending, since about 70% of hospital budget amounts to employees’ salaries. Besides that, the state finances only 80% of health care services in the public sector while the European average is 90% (7).

The functioning of 64 hospitals in Croatia and policy of employment in health has to be urgently defined.

Third, spending in health should be made public and completely transparent.

Fourth, the consequences of the conflict of interest in health should be urgently analyzed (dual practice, the relationship between physicians and pharmaceutical industry, public procurement) and these consequences have to be reduced by adequate regulations.

Also, financial results of supplementary payments in health care (spending in supplementary insurance, effects of co-payments) should be analyzed and a more compact and more just system of co-payments should be made by taking into consideration that more out-of-pocket spending comes from the poor than from the rich part of the population (8).

The implementation of the Law on Patients’ Rights should be revised by abolishing national and local committees and introduce independent institutions (ombudsman) in health care institutions.

Compensation funds for the unwanted treatment outcomes and physicians’ mistakes should be established in the public health sector.

The real reasons for the delays in providing medical care (waiting lists) should be determined and dealt with.

Private health care market should be regulated and the relationship with the public sector established (paying of taxes, fulfilling its responsibilities to the Croatian Institute for Health Insurance, quality control, etc).

Cumulative work time (double salary for clinical university teachers, from hospitals and universities) should be regulated, which is not the case at the moment.

The members of all medical committees in the country should be made known and their work should be made transparent at all times.

An expertise on the work of health centers after its semi-privatization in the past should be made, data should be publicly presented, and a viable solution offered. Mentioning of Andrija Štampar is completely inadequate for today’s public health, organizational, and financial conditions.

If someone would indeed undertake all these measures, the system would fall under the definition of a public system instead of ‘spilling into’ the private sector.

Only then we can talk about individual health care measures: medicines, public-private partnership, payments, insurance, amortization, changes to bioethical laws, etc.

Expenditures for carrying out such changes are minimal and involve switching from a bureaucratic system to the one interested in important functions.

However, the consequence of these changes would be the loss of the privileges of financial and professional irresponsibility for most of the people employed within the system. This problem is difficult to overcome since all the individuals who come to the minister’s position are a part of the system. Therefore, it is difficult expect that the current health, political, and professional circles could produce a person who could make serious changes to the health care system.

What is expected is that instead of continuing with the policy of ‘fixing’ the incidents we go back to the beginning, analyze the causes, and reorganize the system by means of the instruments which are intrinsic to it, rather than the instruments that belong to other fields, such as economy.

I am afraid that the conclusion is that it is too late and that the Croatian health care will remain to be perceived by the politicians and the public as a grim alternative to an expensive, polished, comfortable private system that sells goods of questionable quality. The only hope is that the citizens and civil society will understand that they have rights because they finance this system and that they will start insisting on their realization. However this could be a slow and extremely painful process.

## References

[1] Social Democratic Party. SDP Healthcare Strategy [in Croatian]. Available from: http://www.sdp.hr/politike/zdravstvo Accessed: June 8, 2011.

[2] Anonymous. Patients against dual practice for physicians [in Croatian]. Available from: http://www.index.hr/vijesti/clanak/pacijenti-protiv-privatnog-rada-lijecnika-u-bolnicama/266574.aspx Accessed: June 7, 2011.

[3] Babić J. Former minister in scandalous nomination [in Croatian]. Available from: http://www.nacional.hr/clanak/10151/skandalozna-kandidatura-bivse-ministrice Accessed: June 8, 2011.

[4] Croatian Radio Television. Deputy health minister resigns [in Croatian]. Available from: http://www.hrt.hr/arhiv/2002/02/01/HRT0046.html*.* Accessed: June 9, 2011.

[5] Tolić T. Who has earned from the procurement of CT scan for Clinical Hospital in Zagreb? [in Croatian]. Available from: http://www.monitor.hr/clanci/tko-je-zaradio-22-milijuna-kuna-na-kupnji-ct-uredaja-za-kbc-zagreb/15241/ Accessed: June 5, 2011.

[6] Škaričić N. Physicians in service for pharma-industry [in Croatian]. Available from: http://www.hsp1861.hr/vijesti4/030710ns.htm Accessed: June 6, 2011.

[7] Stavljenić-Rukavina A. Vision of health care politics through 10 questions [in Croatian]Hrvatski Časopis za Javno Zdravstvo 2007Oct 73

[8] Mastilica M, Kušec S (2005). Croatian healthcare system in transition, from the perspective of users.. BMJ.

